# Sebaceous Carcinoma Masquerading As Orbital Cellulitis

**DOI:** 10.7759/cureus.22288

**Published:** 2022-02-16

**Authors:** Vignesh Ramachandran, Gayane Tumyan, Asad Loya, Kristina Treat, Ivan Vrcek

**Affiliations:** 1 Internal Medicine, Texas Health Presbyterian Hospital, Dallas, USA; 2 Dermatology, New York University, New York, USA; 3 Ophthalmology, Baylor College of Medicine, Houston, USA; 4 Pathology, Texas Health Presbyterian Hospital, Dallas, USA; 5 Oculoplastics, Texas Eye Plastics, Dallas, USA

**Keywords:** delayed diagnosis, malignancy surgery, inpatient care, medical resident education, orbital cellulitis, sebaceous carcinoma

## Abstract

Sebaceous cell carcinoma is an uncommonly encountered cutaneous malignancy. Often considered a great masquerader, sebaceous cell carcinoma arises from meibomian glands and can have a poor prognosis if not diagnosed early. In this case report, we present a patient with sebaceous cell carcinoma who presented to our emergency department with a clinical presentation that was concerning for orbital cellulitis. The patient was initially started on intravenous antibiotics. However, workup, including imaging and laboratory results, pointed toward malignancy as the diagnosis. The patient underwent an incisional biopsy and pathology confirming the diagnosis of sebaceous cell carcinoma. We engaged in further discussion of this peculiar cutaneous masquerader, differential diagnoses, and important considerations.

## Introduction

Sebaceous cell carcinoma is a rare, potentially lethal cutaneous malignancy often arising from the eyelids given its plethora of meibomian glands. Although sebaceous cell carcinoma represents the third most common malignant eyelid neoplasm after basal and squamous cell carcinomas, it only accounts for approximately 0.5% of eyelid tumors (benign or malignant) [1]. Herein, we present a case of sebaceous cell carcinoma masquerading as orbital cellulitis.

## Case presentation

The patient is a 78-year-old female with hypertension, major depressive disorder, generalized anxiety disorder, and gastrointestinal reflux disease who presented to our hospital with left-sided vision loss for several weeks. Overall, her symptoms initially started with periorbital swelling and pain starting four months prior. Fearful of the ongoing COVID-19 pandemic, she delayed seeking medical care until her symptoms became intolerable and eventually resulted in loss of left-sided vision. She denied fevers or chills but did endorse decreased appetite and unintentional weight loss. She denied periorbital trauma. Family and social histories were not contributory. 

On admission, the patient was afebrile and had stable vital signs. The physical examination revealed left periorbital edema, erythema, warmth, and tenderness to palpation. Two separate 2 cm enlarged left-sided parotid and submandibular masses were also present. She was able to count fingers with her right eye (distance visual acuity was not tested), and her visual acuity was limited to merely light perception in the left eye. Extraocular movements were limited by pain in the left eye.

**Figure 1:**
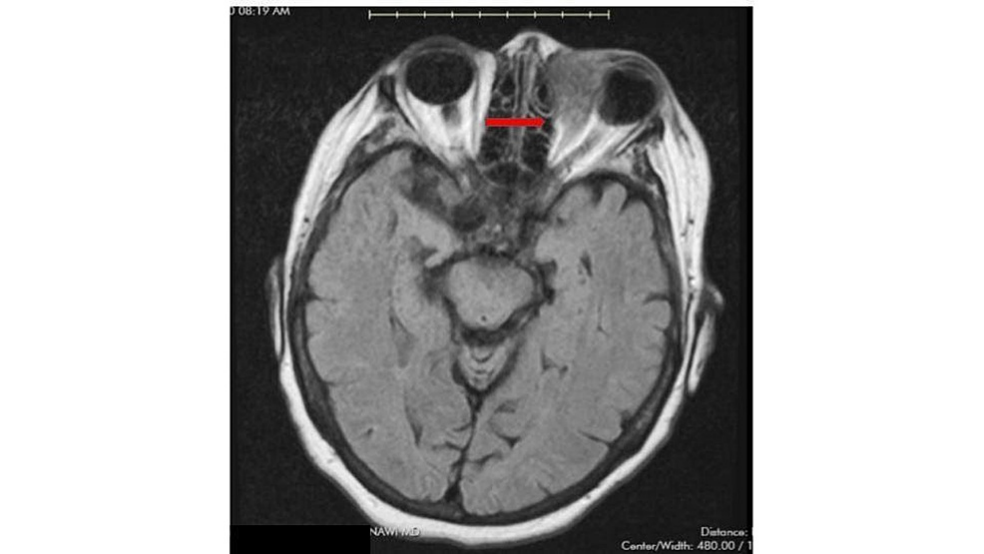
MRI revealing heterogeneously enhancing extraconal mass within left orbit infiltrating into the medial canthus region (red arrow).

She was empirically started on intravenous vancomycin and cefepime in the emergency room for concern for orbital cellulitis while awaiting magnetic resonance imaging studies (face/neck/orbits/brain). A prompt ophthalmology consult was done for the same. Imaging revealed a heterogeneously enhancing extraconal mass within the left orbit, measuring 3.5 x 3.5 x 3.2 cm, infiltrating into the soft tissue structures of the eyelids and medial canthus region (Figure 1). Additionally, there were also intraparotid lesions and bulky left cervical lymphadenopathy.

**Figure 2:**
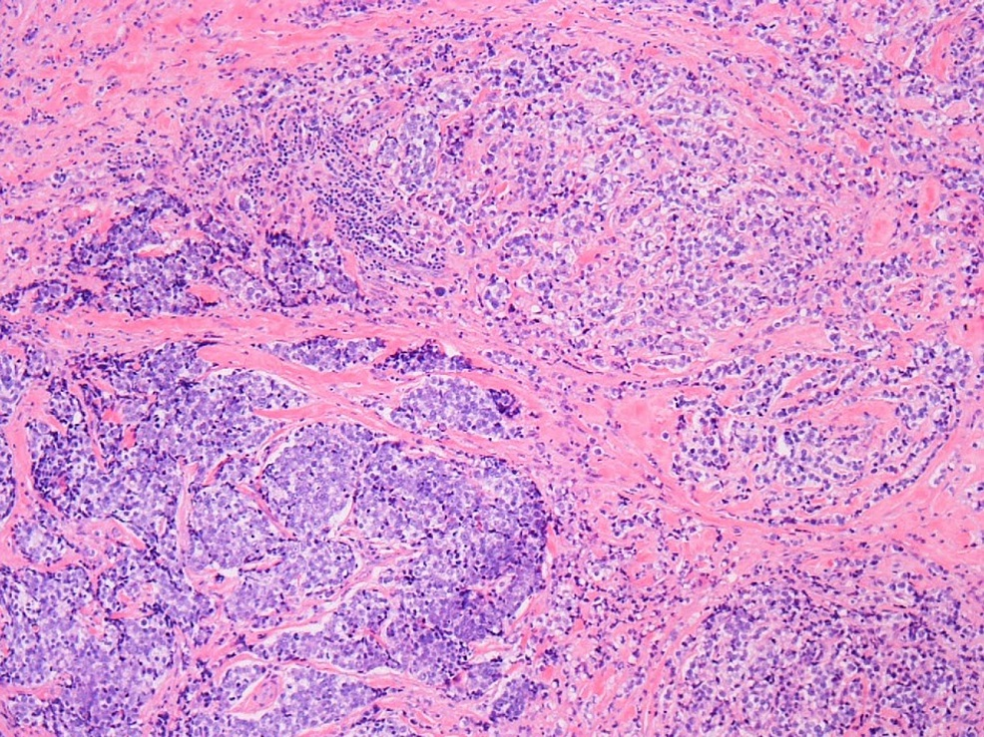
Surgical pathology from the intraocular specimen resected in the operating room revealed a specimen consisting of fibrous tissue with nests, sheets, and cords of moderately pleomorphic small to medium-sized tumor cells.

**Figure 3:**
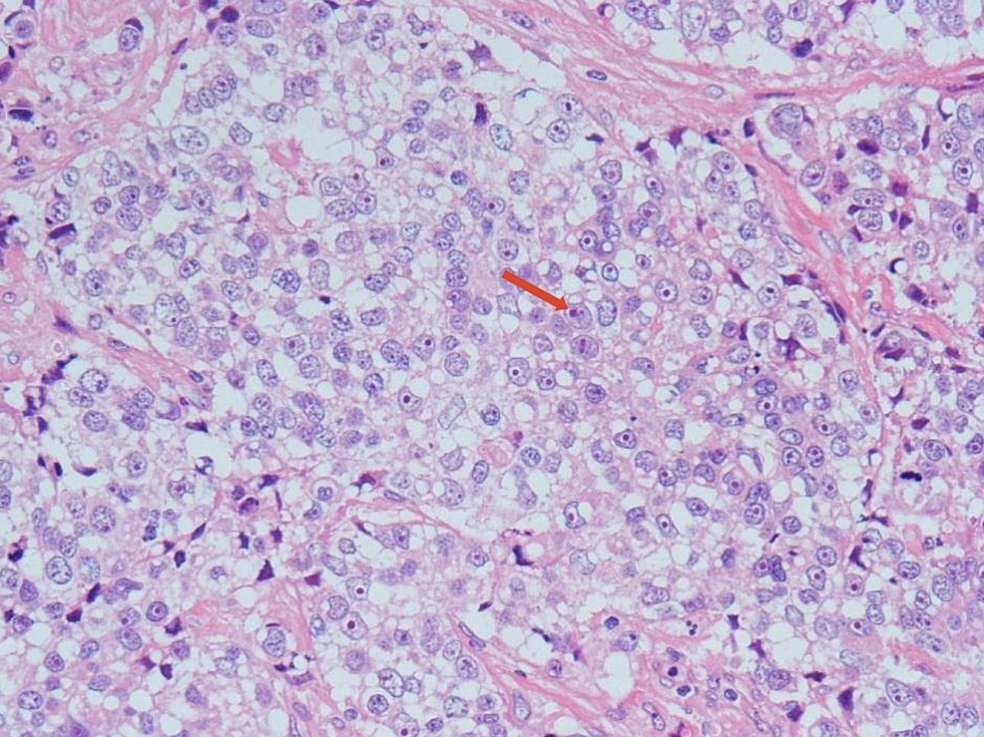
Surgical pathology from the intraocular specimen resected in the operating room revealed focal areas of the tumor showing larger vesicular nuclei with one or more eosinophilic nucleoli and more abundant multivacuolated cytoplasm (example highlighted with red arrow). The vacuolated cytoplasm, along with the presence of pagetoid spread, is pointing toward the diagnosis of sebaceous carcinoma. No mature appearing sebaceous cells were identified.

**Figure 4:**
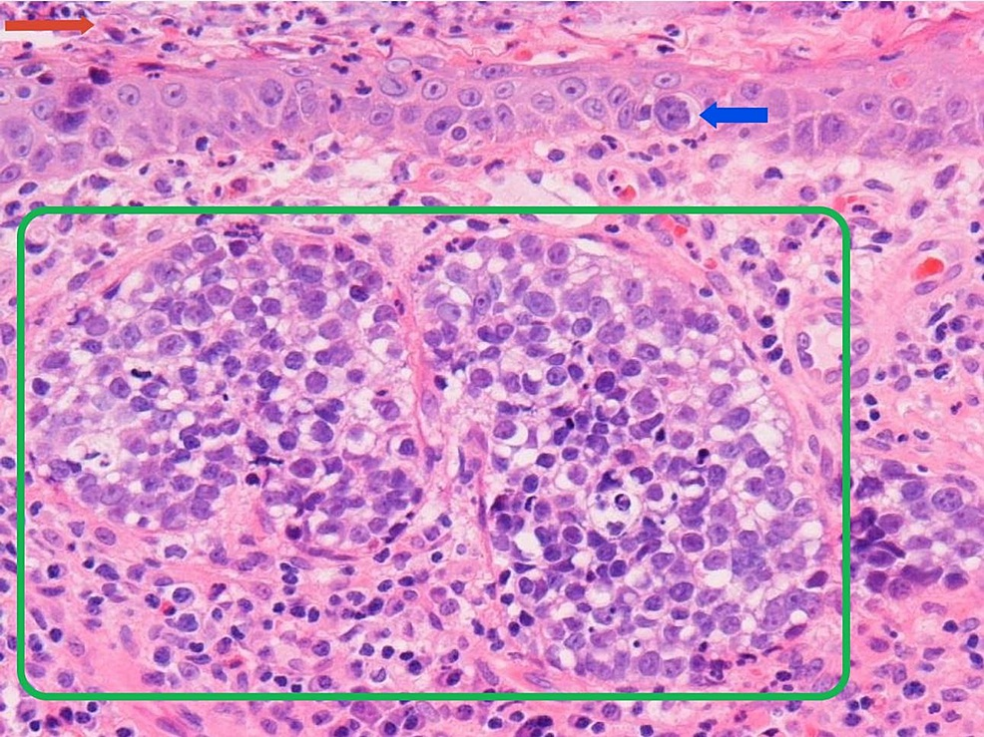
One of the fragments contained focal squamous epithelium (at the top, horizontal region labeled with red arrow). A few nests of tumor cells with focal vacuolated cytoplasm were present underneath this epithelium (green box), with focal intraepidermal (pagetoid) seen (blue arrow highlighting a classical example of a pagetoid cell). The pagetoid infiltration of the overlying epithelium is another clue to the diagnosis, as it is commonly observed in periocular sebaceous carcinoma.

**Figure 5:**
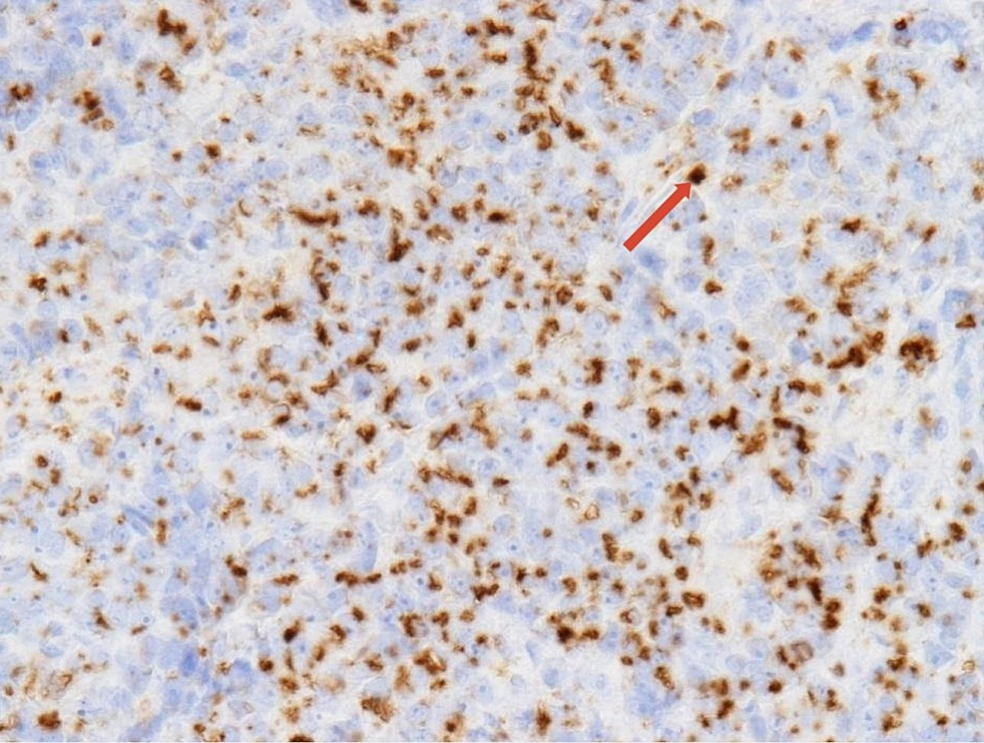
The diagnosis was confirmed with the positive membranous vesicular staining for adipophilin (dark staining highlighted with the red arrow).

The patient underwent an orbitotomy of the left eye with pathology revealing a working diagnosis of presumed metastatic sebaceous cell carcinoma (Figures 2-5). Metastatic disease was proposed given the pathology of the tumor and imaging findings of enlarged regional lymph nodes (evidence of local tumor extension) and extensive left-sided lymphadenopathy with multiple large enhancing lesions within the left parotid gland (the most common location for metastasis of sebaceous carcinoma). Further staging, including biopsy and positron emission tomography, was deferred to the outpatient setting.

Subsequent imaging of the abdomen and pelvis revealed wall thickening of the gastric antrum with adjacent subcentimeter mesenteric lymph nodes. Gastroenterology was consulted and endoscopy revealed peptic ulcer disease with pathology confirming this diagnosis, ruling out additional sebaceous cell tumors that may be associated with Muir-Torre syndrome.

## Discussion

Sebaceous cell carcinoma was first described more than a century ago by Fuchs [2]. The incidence of sebaceous carcinoma varies widely depending on ethnicity and geographical region. While uncommon among white patients, it is more frequently encountered in the Asian population [3].

Establishing the diagnosis of sebaceous cell carcinoma may be difficult for both the clinician and the pathologist because of its myriad of presentations. The tumor can frequently present as a hard, immobile nodule located in the upper tarsal plate that may have a multicentric origin. Other common manifestations include diffuse thickening of eyelids associated with loss of eyelashes [4]. 

Sebaceous carcinoma is more common in elderly patients, with a mean age of diagnosis in the mid-sixties [4,5]. Younger patients are thought to be at risk if there is a history of radiation, immunosuppression, or hereditary cancer syndromes [5]. Local and distant metastases are common and are dependent on the location and size of the primary tumor [6]. The most common locations for distant metastasis of sebaceous carcinoma are the liver, lungs, brain, and bones [7].

Local tumor invasion and extension into the orbit can mimic inflammatory conditions. Sebaceous cell carcinoma may also masquerade as other inflammatory states such as chronic unilateral blepharoconjunctivitis or chalazion [8,9]. Atypical presentation of these conditions or lack of response to appropriate treatment may be concerning for sebaceous carcinoma. Other important malignancies to consider in the periorbital region are ocular lymphomas, squamous and basal cell carcinomas, metastases, and Merkel cell carcinomas. Additionally, Muir-Torre syndrome, an autosomal dominant condition with various sebaceous-type malignancies of the gastrointestinal tract, urologic, and endometrial tissues, should be considered [10].

Exposure to ophthalmology is limited for internal medicine residents [11]. While hospitalists and internal medicine residents may be familiar with acute infectious ocular pathologies, such as periorbital cellulitis, malignancies, such as sebaceous cell carcinoma, may be encountered less often. To prevent anchoring bias, a deliberate history and exam can broaden the differential. This is especially important in our case as sebaceous cell carcinoma is referred to as “the great masquerader” with studies revealing a significantly worse prognosis with a delayed diagnosis [12].

Additionally, it is noteworthy that our patient’s care was substantially delayed due to the COVID-19 pandemic. Disease entities that can present like a chalazion or other benign conditions may have delayed evaluation and recognition until metastasis has occurred in the current COVID-19 environment. It is important to provide healthcare via telemedicine or home health visits, especially to patients who are hesitant to visit hospital/clinic due to COVID-19 pandemics to prevent irreversible sequelae. 

## Conclusions

A difficult to diagnose malignancy, sebaceous cell carcinoma, arises from meibomian glands and can have a poor prognosis if not diagnosed early. Establishing the diagnosis of sebaceous cell carcinoma is difficult. This is exacerbating by the lack of exposure to ophthalmology that medical students and residents in internal medicine receive. In this day and age of a global pandemic during which patients are afraid to enter hospitals, entities that can present like a chalazion or other benign conditions may have delayed evaluation and recognition until metastatic disease has occurred.
